# Redox Profiling of Selected Apulian Red Wines in a Single Minute

**DOI:** 10.3390/antiox11050859

**Published:** 2022-04-27

**Authors:** Douglas Vieira Thomaz, Renê Oliveira do Couto, Riccardo Goldoni, Cosimino Malitesta, Elisabetta Mazzotta, Gianluca Martino Tartaglia

**Affiliations:** 1 Department of Science, Biological and Environmental Technology, Università di Salento, 73100 Lecce, Italy; cosimino.malitesta@unisalento.it; 2 Midwest Campus Dona Lindu, Federal University of Sao Joao del-Rei, Divinopolis 35501-296, Brazil; rocouto@ufsj.edu.br; 3 Department of Medicine, Surgery and Dentistry, Università Degli Studi di Milano, 20122 Milan, Italy; riccardo.goldoni@polimi.it; 4 Maxillofacial and Dental Unit, Policlinico di Milano, 20122 Milan, Italy

**Keywords:** oxidation, redox, nutraceutical, antioxidant, wine, multivariate, fingerprint

## Abstract

Wine is a complex bioproduct whose chemical composition is highly variable across production regions. In order to shed light on affordable ways to promote the characterization of wines and explore the physicochemical basis of their antioxidant capacity, this work reported on the quick and easy redox profiling of selected red wines from Apulia, Italy. Therefore, an affordable and quickly performed semiempirical quantum chemistry approach, i.e., the extended Hückel method, was used to compute the bandgaps of the main phytochemical markers attributed to red wines. The findings of these calculations were then compared to an electroanalytical investigation in the form of cyclic and square-wave voltammetry, and the electric current of the redox profiles was used as the input dataset for principal component analysis. Results showcased that the semiempirical quantum chemistry calculations allowed the correlation of the bandgaps to the observed faradaic signals upon voltammetry; thereby, also providing insights on their antioxidant appeal by highlighting the feasibility of charge-transfer processes at low electric potentials. Furthermore, the principal component analysis showed that the electric current dataset gathered in a time span of 55 s allowed the appropriate separation of the samples, which hints at the possible use of quick voltammetric assays as fingerprinting tools.

## 1. Introduction

Wine is a complex bioproduct whose chemical composition is highly variable according to edaphologic and production features. Nonetheless, the intrinsic physicochemical nature of the soil and the characteristics of the regional climate are known to play major roles in the organoleptic profile of wine grapes, as well as in the composition of their secondary metabolites [1]. In fact, several authors detailed the effects of soil organomineral content, acidity and alkalinity, moisture, temperature, as well as other factors in the stimulation of the phenylpropanoid metabolic pathway in grapes and other plants [2], thereby shedding light on the environmental influences on the aroma and mouthfeel of wines, as well as contributing to their chemical profiling according to geographic origin.

Concerning the geographic origin, wines may be amenable to protected designation of origin status [3], which is granted upon the combination of particular sensorial features and traditional production methods which allow their grouping according to a restricted sourcing area [4]. Therefore, even though the designation may hint at standardization, wines from the same protected designation may showcase very distinct organoleptic profiles. In this regard, several authors have proposed the metabolomic investigation of grapes and wines to allow their systematic classification according to chemical composition [5]. Nevertheless, these studies are highly expensive and time-consuming, what severely hinders their applicability.

In regards to the chemical composition of wines, literature states that polyphenolic secondary metabolites are the most representative class of phytocomponents in this product [6]. In fact, phytochemical markers such as flavan-3-ols [7], flavonols [8,9], anthocyanins [10], stilbenes [11] and phenolic acids [12] are well reported to be found in ubiquity in red wines. Moreover, several mevalonate and phenylpropanoid derivatives have been linked to wine aroma (i.e., volatile phenolic acids), color (i.e., anthocyanins) and mouthfeel (i.e., tannins) [13]. The presence and diversity of phenolic compounds in wine is attributed not just to the grape type and variety, but also to the complex reactions that the chemicals in the grape must undergo during fermentation [14]. In this regard, owing to the highly particular chemical composition of wines, chemical fingerprinting analysis would be highly beneficial in detecting frauds that could, otherwise, lead to substantial financial losses [15,16].

It is noteworthy that over the last few years, the very same constituents that could be used in the chemical fingerprinting of wines have been extensively investigated in regards to their putative health benefits. For instance, in the context of oral health, it has been suggested that moderate red wine consumption may help to avoid gum ailments such as periodontal disease [17], which is an inflammatory ailment of the tissues of the periodontium that has high prevalence (up to 90%) in elderly people [18]. Likewise, photoactive antioxidant compounds such as anthocyanins, as well as their aglycone counterparts, the anthocyanidins (i.e., cyanidin), are known to not only provide the red-bluish hue of wines, but also exhibit strong cardioprotective, vasorelaxant and anti-inflammatory activities in vivo [19].

Although the chemical profiling of wines by means of refined methods such as mass spectrometry may be hindered due to financial constraints, alternative approaches may allow an overview of selected physicochemical features upon limited investment of equipment and infrastructure. In this regard, many authors have cited electrochemistry in tandem with computational methods to explore the chemistry of complex samples and characterize them [20]. Nonetheless, the redox profiling and the investigation of the voltammetric behavior through cyclic and square-wave voltammetry have been reported as being highly affordable and straightforward ways to promote the redox fingerprinting of foodstuff and bioproducts [21], thereby leading in some instances to the development of authentication tools against counterfeiting [22]. Moreover, owing to the charge-transfer basis of the therapeutic properties tied to wine bioactive compounds, the use of electrochemistry allows a comprehensive overview of the underlying kinetics and thermodynamics of antioxidant capacity [23,24].

Owing to the relevance of the physical chemistry of natural products in their surrogate nutraceutical applications, some researchers have also proposed the use of theoretical chemistry to predict their behavior [25]. In this sense, ab initio quantum chemistry approaches were used to shed light on the energies of the highest occupied molecular orbital (HOMO), as well as the lowest unoccupied molecular orbital (LUMO) in order to calculate the bandgap between frontier orbitals [26]. Nonetheless, the most often-reported approach to achieve this information is the highly refined density-functional theory (DFT), which proves itself as a suitable and versatile tool to explore not just the energy levels, but also the molecular topology of complex systems. However, the use of DFT needs the screening, selection and use of adequate terms for the calculations, as well as requiring a great deal of computational power and long running times [27,28], which led several authors to adopt the use of semiempirical methods that require dramatically less computational power [23,29,30].

By all accounts, the estimation of bandgap energies allows the exploration of the thermodynamic feasibility of charge-transfer processes, which are one of the key features of antioxidant capacity. In addition, the combination of theoretical analysis with empirical evaluation through electrochemistry allows researchers to correlate specific sets of signals in voltammetric analysis with selected groups of structurally similar electroactive compounds, which greatly assists in gathering information about the reducing power of the sample. As such, systematically combining these physicochemical methods could lead to the facile characterization of the redox behavior of complex samples, without having to resort to spectrophotometric assays based on free-radical scavenging methods or colorimetric evaluations.

In this sense, in view of its relevance in shedding light on affordable ways to promote the redox characterization of wines, as well as exploring the physicochemical basis of their antioxidant capacity, this work reports the redox profiling of selected red wines from the region of Apulia, Italy, by means of electrochemical and computational approaches.

## 2. Materials and Methods

### 2.1. Samples and Electrolytes

The samples consisted of six varietal red wines; all were of protected designation of origin as disclosed by the producers. The wines were produced by local winemakers in the neighboring areas of the municipality of Lecce (LE). The samples were coded and labeled according to the following: primitivo gioviale (A); primitivo secco (B); primitivo amabile (C); merlot (D); malvasia (E); and negroamaro barricato (F). The vintage of all wines was 2021; all of them except sample F were stored in INOX tanks at room temperature. Sample F was stored in wooden caskets of undisclosed nature by the producer. The samples were retrieved from their sources and tested within the maximum timespan of 48 h.

The protected designation of origin of the Primitivo variety of the samples herein selected was the Primitivo di Manduria, also known as Zinfandel, which is a highly tannic grape whose wines are full-bodied and distinctively aromatic. Primitivo di Manduria is genetically similar to the Croatian Crljenak grape variety, and has been a staple product of Taranto for many years [31]. Likewise, the Negroamaro variety is also strongly linked to the Apulia region, especially in the neighboring areas of Alezio, Brindisi, Copertino, Galatina, Gioia del Colle, Lizzano, Leverano and Terra d’Otranto; it is used to produce full-bodied and aromatic wines [32]. On the other hand, Malvasia Nera, a wine variety of remarkable dark-red coloration, is under protected designation of origin in the wine-producing areas of Lizzano, Matino, Nardò, Squinzano and Terra d’Otranto [33]. In addition, the Merlot variety, which is known by its medium-bodied profile, is under protected designation in the region of San Severo [33].

### 2.2. Selection of the Commonly Reported Antioxidants in Red Wine

In this work, the following polyphenolic markers were proposed according to previous reports regarding phytochemical analysis of biologically relevant compounds in varietal red wines: catechin as a representative of the flavan-3-ols [7]; quercetin as a representative of the flavonols [8]; cyanidin as a representative of the anthocyanins [10]; resveratrol as a representative of the stilbenes [11]; and gallic acid as a representative of phenolic acids [12]. Their isomeric simplified-molecular-input-line-entry system (SMILES) was gathered from the Pubchem database and used in the theoretical studies. The chemical structures, as well as their HOMO mappings and deposit codes (PubChemID) are disclosed in Table 1.

It must be considered, however, that fermented beverages of nutraceutical appeal such as wine are highly complex samples, and a plethora of compounds, both from primary and secondary metabolism of the particular grape varieties, as well as the fermenting microbiota, will be present in the final product. Therefore, we herein selected compounds that are reported to be ubiquitously distributed in red wines, and are well acknowledged to be the main contributors to the nutraceutical properties of this product.

### 2.3. Energy Refinement by MM2 and AMBER

In order to standardize the handling of cheminformatics data in this work, all selected molecules underwent energy refinement by MM2 and AMBER.

MM2 is a force-field-based method which reliably reproduces the geometry of molecules at equilibrium by implementing a large set of continuously refined parameters, which are updated according to data regarding individual atoms and classes of organic compounds [34]. This approach was selected to preliminarily minimize the steric energy of the compounds herein investigated, and was accompanied by the application of AMBER. The minimum root mean square gradient herein used to optimize the structures was 0.010.

AMBER is an energy minimization method which takes into account the summing of all forces acting on the system in order to estimate its potential energy [35]. The mathematical operation detailing the energy refinement provided by AMBER is described as follows.

$$V\left(r^{n}\right)=\overset{}{\underset{i\in bonds}{\sum }}k_{bi}{\left(l_{i}-l_{i}^{0}\right)}^{2}+\overset{}{\underset{i\in angles}{\sum }}k_{ai}{\left(\theta_{i}-\theta_{i}^{0}\right)}^{2}\;\;\;\;\;\;\;\;+\overset{}{\underset{i\in torsions}{\sum }}\overset{}{\underset{n}{\sum }}\frac{1}{2}V_{i}^{n}\left[1+\cos(n\omega_{i}-\gamma_{i}\right]\;\;\;\;\;\;\;\;\;\;\;\;\;\;\;+\overset{N-1}{\underset{j=1}{\sum }}\overset{N}{\underset{i=j+1}{\sum }}f_{ij}\left\{\epsilon_{ij}\left[{\left(\frac{r_{ij}^{0}}{r_{ij}}\right)}^{12}-2{\left(\frac{r_{ij}^{0}}{r_{ij}}\right)}^{6}\right]+\frac{q_{i}q_{j}}{4\pi \epsilon_{0}r_{ij}}\right\}$$

wherein the steric energy is minimized upon the derivative of the potential energy of the system in regards to position, which can be expressed as the summing of all forces acting therein. The summing of the energies of the bonds and angles is approximated to the harmonic force according to Hooke’s law ($k_{bi}$ being the spring constant of the bonds and $k_{ai}$ of the angles); torsions are calculated from a Fourier series; and the non-bonded energy between all atom pairs is calculated from the Van der Waals and electrostatic energies considering the equilibrium distance ($r_{ij}^{0}$) and well depth (ϵ).

### 2.4. Extended Hückel Method (EHM) and Determination of Molecular Orbital Energies

The semiempirical EHM was applied to the proposed phytochemical markers in the wine samples aiming to evaluate the bandgap between orbitals, which can be associated with the thermodynamic feasibility of redox reactions. Given that the nutraceutical properties of wine are associated with antioxidant capacity which nonetheless involves electron-transfer reactions, this methodology allows the proposition of possible electro-oxidation mechanisms being applied to antioxidant compounds, which could thereafter be compared to the voltammetric profiles.

The EHM calculations are based upon the energies of HOMO and LUMO, wherein only valence electrons (*j*) of *n*-numbered orbitals are computed, and their wavefunctions (*ψ*) are considered individually as described below.

$$\psi_{valence}=\psi_{j}\left(n\right)$$

The molecular orbital energies were calculated according to previous reports [36], thereby summing the energy of individual electrons through an off-diagonal Hamiltonian matrix and filling the diagonal of the Fock matrix with parametrized energies, which yield the energy eigenvalues and wavefunction eigenvectors of valence orbitals. In the EHM, each molecular orbital is computed as a linear combination of atomic orbitals, as described below.

$$\psi_{j}=\overset{N}{\underset{r=1}{\sum }}c_{jr}\varphi_{j}$$

wherein *φ* are valence atomic orbitals; *N* stands for the molecular orbital; *c_jr_* are the weighting coefficients.

The energies of the molecular orbitals are calculated from a single-electron Schrödinger equation using a single-electron Hamiltonian (*h_eff_*), as described below.

$$h_{eff}\psi_{j}=\epsilon_{j}\psi_{j}$$

The Hamiltonian herein used as operator is equivalent to the sum of kinetic and potential energies in classical mechanics systems (i.e., total energy); however, it takes into account the time-evolution and energy spectrum of the system, thereby describing the interaction of the electron with the entire molecule.

All calculations were conducted in silico post steric energy minimization by force field and classic molecular-mechanics-based approaches (i.e., MM2 and AMBER), and the energies of the molecular orbitals (*n* = 0) were expressed as the numeric energy gap between HOMO and LUMO in eV (ΔE).

### 2.5. Electrochemical Measurements

The electrochemical investigation was performed with a portable potentiostat/galvanostat, PalmSens, EmStat4 Blue model. The software interface was PSTrace version 5.8 (PalmSens, Houten, The Netherlands). The electrode system consisted of a saturated calomel reference electrode (SCE), a counter platinum electrode and a standard Ø = 3 mm glassy carbon electrode. The reference, working and counter electrodes were purchased from CH Instruments (Tennison Hill Drive, Austin, TX, USA), and the working electrode underwent thorough cleaning with 0.5 µm alumina before the experimental runs; blank assays were performed in PBS solution pH 7.4 to check adequate cleaning. All electrochemical experiments were performed without deaeration at room temperature (25 °C ± 2) in 3.5 mL solutions, being 3.0 mL PBS and 0.5 mL of samples. The samples underwent no pretreatment before analysis.

Cyclic voltammetry (CV) was performed at 50 mVs^−1^ in the electric potential interval of −0.2 to +1.2 V, for six successive scans. The voltammogram (electric current versus electric potential) and chronoamperogram (electric current versus time) were individually extracted and plotted for the first scan of each sample. Moreover, square-wave voltammetry (SWV) was also used for profiling as a supporting technique to minimize the capacitive influence produced by linear scans of electric potential, and was conducted between −0.2 and +1.2 V, with wave amplitude of 50 mV and step of 10 mV. Furthermore, the frequency was set to 20 Hz, and the forward and reverse currents were represented in the SWV plots.

Considering that the electroanalysis of polyphenolic-rich natural products usually involves extensive polymerization of oxidation products at the working electrode surface [21], the integrated charge was therefore calculated using Cottrell’s equation [37]. This treatment is based on the dependence of charge versus time, and allows information to be drawn from the kinetics of the electro-polymerization process, which is also proportional to the concentration of electroactive species in the cell solution. Therefore, the aforementioned six CV runs were performed without surface renewal in-between cycles, and the charge of their chronocoulometric plots was integrated individually.

Qt=2nFAD12Ct12π12+Qdl+nFAΓ

wherein: *Q* stands for the charge, *t* stands for time, *F* is the Faraday constant, *A* stands for the area of the working electrode, *C* is the concentration of electroactive species in the solution, *Q_dl_* is the capacitive charge and *nFAΓ* is the faradaic component of absorbed species (i.e., surface coverage), which is defined according to the following.

$$nFA\Gamma =Q_{\int C}$$

wherein *Q*_∫*C*_ stands for the integrated area of chronocoulometric plot yielded by each voltammetric scan.

In addition, the kinetics of surface coverage by the adsorption of oxidation products was investigated by the use of non-linear curve fitting based on the Belehradek function, which was applied with the assistance of the Levenberg–Marquardt iteration algorithm. This fitting is based on a three-parameter abscissa-shifted power function, which is defined according to the equation below, and has been extensively reported as a general-purpose tool for non-linear fitting in several applications, ranging from physicochemistry to physiology [38,39].

$$f\left(x\right)=a{\left(x-b\right)}^{c}$$

wherein *a*, *b* and *c* stand for the coefficients of the function.

### 2.6. Statistical Analysis

In this work, principal component analysis (PCA) was used to evaluate if the attributes could harness information to allow the differentiation of the observations, namely the wines. The input dataset herein used was the electric current obtained in the voltammograms. The dimension reduction promoted by the PCA employed a correlation matrix and the extraction of the first two PCs, and was graphically represented through a biplot showcasing the placement of the eigenvectors of the electric current values, and the 2D scatter of the scores, i.e., wine sample code, as observational label. All statistical analysis was conducted considering statistical significance to *p* < 0.05, thereby employing a confidence interval of 95%. Furthermore, the continuous variables were normalized within the range of 0 to 1 in order to standardize the contribution of each attribute (discrete value of electric current) to the model.

## 3. Results

The first step in this investigation consisted of evaluating the bandgap between the HOMO and the LUMO of the proposed phytochemical markers of the wine samples. To this end, EHM was used. Results are displayed in Table 2.

Results showed that the bandgap of catechin was 12.178 eV, whilst that of quercetin was 6.183 eV. The bandgap of cyanidin, resveratrol and gallic acid were 4.852, 7.449 and 8.016 eV, respectively (Table 2).

**Figure 1:**
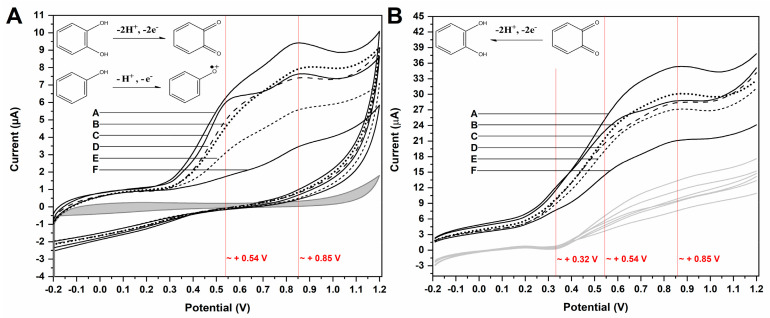
(**A**) CV and (**B**) SWV outputs of each selected red wine sample (A to F). The electric potential associated with the main possible faradaic signals which could be hinted at in the voltammograms is therein highlighted in red. The blank CV signal is represented in gray, and the SWV plot comprises the forward currents of each sample (therein indicated), and the reverse current is in light gray. Moreover, the possible physical origin of the signals is represented by the mechanisms as inset images.

Following this, all wine samples underwent electrochemical investigation through CV and SWV in order to evaluate their redox profile and voltammetric behavior. Results are presented in Figure 1.

Results showed that the voltammetric profile of the wines was distinct. Upon CV, all wines presented two seemingly irreversible anodic processes at *E*(V) ≈ +0.54 and +0.85 V. These processes varied in amplitude and shape, the influence of capacitive currents being very conspicuous, thereby leading to poor signal distinguishability (Figure 1A). On the other hand, the SWV findings showed that all samples presented two anodic processes and one cathodic process at *E*(V) ≈ +0.32 (Figure 1B).

**Figure 2:**
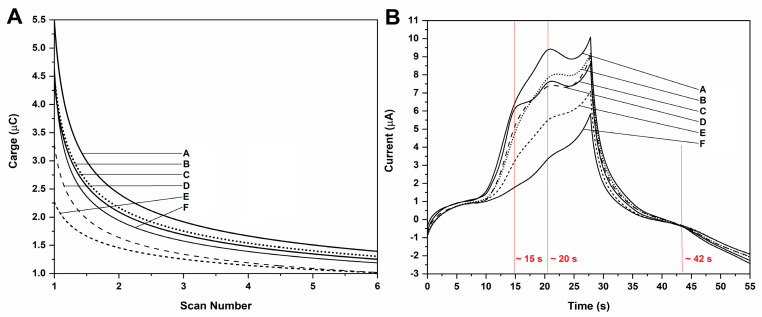
(**A**) Integrated charge of the chronocoulograms fitted according to Belehradek function (all fittings above r^2^ 0.98). (**B**) Chronoamperograms extracted from CV scans. The samples are therein indicated (A to F). The time associated with the visualization of each faradaic process is highlighted on the chronoamperogram with a red color.

After CV analysis, the integrated charge of the chronocoulograms was plotted against the scan number (*n* = 1 to 6), and the chronoamperogram was extracted to validate the observations made through CV, as well as to check the time at which the signals were visible upon a scan rate of 50 mVs^−1^. Results are presented in Figure 2.

Results evidenced that the kinetics of charge-transfer upon increasing scan numbers follows an exponential trend which is adequately described by a power function (Figure 2A). Moreover, the same faradaic processes and capacitive contributions which were observed during CV were visible in the chronocoulogram. The timestamps of each of these processes were determined as: 15 s and 20 s for the two anodic processes at *E*(V) ≈ +0.54 and +0.85 V, respectively; and 42 s for the cathodic process at *E*(V) ≈ +0.32 (Figure 2B). The total time required to perform the redox profiling by CV was 55 s for each sample.

**Figure 3:**
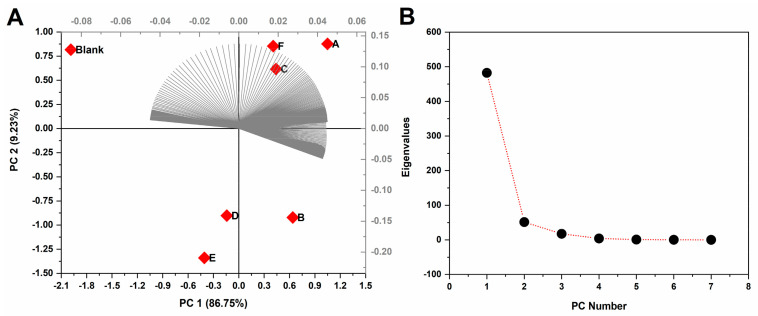
(**A**) PCA biplot of the electric current of the first CV scan of each of the samples (label variables in red). The amount of variance explained by each PC is therein informed in percentage, and the eigenvectors of the discrete electric current values is therein represented in gray. (**B**) Plot of the eigenvalues versus PC number, which was used to refine the PCA.

Thereafter, the single-minute data of the electric currents from each first CV scan of the samples were inputted into a PCA. This was performed in order to test if the electric current associated with the redox profile of the samples garnered enough information to allow their differentiation. Results are presented in Figure 3.

The results of the PCA showed that the first PC accounted for 86.75% of all variance in the model, while the second PC amassed 9.23%. The total variance explained by the PCA was 95.98%, thereby hinting at a reproducible model with the single-minute voltammetric data (Figure 3A). The plot of the eigenvalues versus PC number indicated that the first PC did indeed account for most of the variance in the model, and subsequent PCs after the second would not meaningfully contribute to a model. Therefore, the selection of the first two PCs was deemed adequate (Figure 3B). Moreover, the eigenvectors of the discrete electric current values showcased an ordered spread on PC1 and PC2.

## 4. Discussion

The presence of polyphenolic compounds in wine is widely reported, and several authors have correlated the consumption of these chemicals with the nutraceutical benefits of this product. In fact, the main representative of flavan-3-ols, i.e., catechin, as well as the main representative of the flavonols, i.e., quercetin, were found to exhibit strong antioxidant capacity and anti-inflammatory activities in vitro and in vivo [40]. Nonetheless, the potential of these markers to scavenge reactive oxygen species has often been associated with the therapeutic benefits of wine consumption in evidence-based medicine, and many authors have reported moderate intake in the prophylaxis of neurodegenerative diseases such as dementia and other illnesses linked to oxidative stress [41].

Overall, the aforementioned therapeutic properties of wine’s secondary metabolites can be correlated to their potential to scavenge reactive oxygen and nitrogen species in the biological medium, thereby decreasing oxidative stress [30,42]. In fact, several authors highlighted the strong antioxidant capacity of wines using free-radical scavenging methods [43], thereby evidencing the role of antioxidant capacitive properties in the nutraceutical properties of wine. Nevertheless, the underlying physicochemistry of these processes has not been as frequently explored in literature as profiling tools.

Considering that the bandgap between frontier orbitals is lower, charge-transfer processes are more thermodynamically feasible [44]; it can be suggested that the order of reactivity of the selected antioxidant markers is (from higher to lower feasibility): cyanidin; quercetin; resveratrol; gallic acid; and catechin (Table 2). Although catechin, quercetin and cyanidin are structurally similar, catechin presented the highest bandgap and is seemingly the most stable of the compounds herein evaluated, as more energy is needed to promote electron transfer between the frontier orbitals. On the other hand, quercetin, cyanidin, resveratrol and gallic acid exhibited lower energies, which could be attributed to the participation of all carbons in the conjugated bonds across the structures [45]. In fact, the lowest bandgap of cyanidin hints that the positive charge of the chromelynium ion contributes to the delocalization of the π electrons, thereby making it more feasible for the molecule to undergo redox reactions, which is nonetheless corroborated by literature [44].

It was noteworthy that the bandgaps of resveratrol and quercetin were strikingly similar to those reported by other authors [46], whilst that of cyanidin to a large degree overlapped with results of DFT at the CAM-B3LYP/6-31+G(d,p) level of theory [44]. Moreover, it has been reported that structurally similar anthocyanins also exhibit similar bandgaps to those herein attributed to cyanidin, such as, for instance: delphinidin (4.8166 eV), peonidin (4.6925 eV) and cyanin (4.9414 eV) [44]. These findings therefore highlight the reliability of EHM when confronted with highly refined ab initio quantum chemistry calculations.

Although other authors have employed highly refined methods such as quantum-chemistry-based DFT to explore the energy levels and molecular topology of natural and synthetic products [47,48], the sheer cost and time-consuming nature of this strenuous computational method hinder the widespread use of ab initio techniques in all areas of chemistry. Therefore, considering the known reliability of semiempirical approaches, the use of EHM in this work showcases a remarkably straightforward way to investigate the thermodynamic feasibility of oxidation in constrained models, such as the sp^2^-hybridized domains of the selected compounds.

The thermodynamic feasibility of polyphenolic compounds readily undergoing charge transfer processes has been extensively discussed in literature as a contributing factor for their acknowledged antioxidant appeal [49]. In fact, the oxidation of phenolic moieties is known to require small disturbances in the reaction medium (i.e., application of electric potentials at *E*(V) ~ +0.5), which can lead to a variety of effects, such as free-radical scavenging in organisms, or restitution of endogenous antioxidant enzymes [41]. In this regard, the narrow bandgaps exhibited by the selected phytochemical markers herein investigated can be associated with the higher feasibility of these compounds behaving as reducing agents [46]. Moreover, owing to the low values of the faradaic processes observed upon CV and SWV, it is reasonable to correlate them with redox transitions in wine antioxidant markers.

Considering that upon behaving as reducing agents, the catechol moiety of polyphenols is converted to a quinone, it can be suggested that the proposed phytochemical markers would follow a similar redox mechanism, which has as a main feature the equivalence of the number of transferred electrons and protons [50]. The presence of catechol-quinone reversible systems in products with vegetal origins has been reported by many authors in electroanalytical investigations, and is further corroborated by the electro-accumulations in the HOMO mapping (Table 1), thereby hinting at the role of these regions in the charge-transfer reaction [23]. Nonetheless, this observation led us to propose the participation of these moieties in the electro-oxidation and electro-reduction reactions that sourced the faradaic outputs observed in the voltammetric assays (Figure 1 and Figure 2). Moreover, the formation of catechol-quinone systems has not only been correlated to antioxidant capacity but has also been presented as a means to establish sample identification through voltammetric fingerprinting [21].

Overall, the CV and SW voltammograms allowed the visualization of the distribution of the faradaic processes; however, as the modulation of electric potential in profiles other than linear functions is known to minimize capacitive influences mid-analysis, SWV allowed clearer signal observation [51]. In addition, given the drop-in signal amplitude according to each CV scan, it can also be hinted that electro-oligomerization may take place on the working electrode surface, which makes capacitive influences more conspicuous. This interpretation is further corroborated by other reports detailing the formation of non-conductive polymer chains atop carbon electrodes that block charge transfer, thereby leading to fouling [52]. The polymerization of phenols is a known phenomenon in the electroanalysis of natural products, as well as in wine production, as it is involved in the change in organoleptic properties upon aging, such as paler color and smoother taste [53]. Nonetheless, the integrated charge of the chronocoulometric plots evidenced that the adsorption of oxidation products leads to the increase in the surface coverage of the working electrode and consequent decrease in signal output (Figure 2).

To all purposes, the wine samples showcased distinct profiles upon electrochemical investigation, and their signals were differentiated enough to lead to their separation in the PCA (Figure 3). As such, the observational labels representing the samples in the PCA showcased separation; the blank was separated from all observations, which was an expected finding, and was herein used as control. On the other hand, the datasets of electric current allowed the visual separation of the samples into seemingly two groups, one comprising samples A, F and C, and another comprising samples B, D and E. Nevertheless, this visual separation could lead to wrong inferences, as PC1 contributed almost ten-fold more to the loadings. In this regard, it is more reasonable to assume that the samples could be listed according to their projections on the abscissa axis, hence: samples E and D, followed by F, C, B and A. This separation is seemingly reasonable, as the Merlot and Malvasia varieties are known to differ chemically and organoleptically from the Primitivo and Negroamaro varieties [31,32,33]. Notwithstanding, owing to the constrained dataset provided by only six varieties, we intend to expand our findings as a preliminary study, and more studies will be followed with larger sampling in order to fully validate the single-minute redox characterization method herein described.

## 5. Conclusions

This work reported the redox profiling of selected Apulia red wines in order to promote insights into their antioxidant capacity and voltammetric behavior. Results showcased that the semiempirical quantum chemistry calculations allowed the correlation of the bandgaps to the observed faradaic signals upon voltammetry. Moreover, PCA showed that the electric current dataset gathered within a time span of 55 s allowed the appropriate separation of the samples, which hints at the possible use of quick voltammetric assays as fingerprinting tools.

## Figures and Tables

**Table 1:** Selected ubiquitous antioxidant markers in wine, as well as their structures, HOMO mappings and PubChemID. The electro-accumulation regions in the HOMO mapping are showcased in dark gray and red color, accounting for positive and negative charges, respectively. All atoms and bonds are colored in black to aid visualization.

Compound	Chemical Structure	HOMO Mapping	PubChemID
Catechin	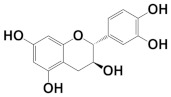	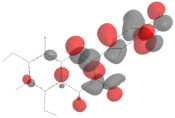	9064
Quercetin	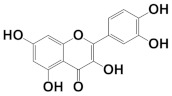	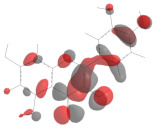	5280343
Cyanidin	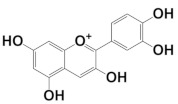	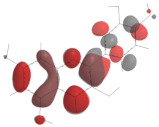	128861
Resveratrol	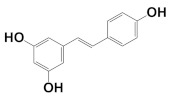	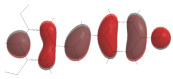	445154
Gallic acid	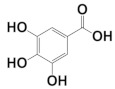	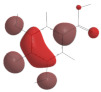	370

**Table 2:** Bandgap in eV of the proposed phytochemical markers in wine.

Compound	HOMO (eV)	LUMO (eV)	ΔE (eV)
Catechin	−10.885	1.293	12.178
Quercetin	−10.086	−3.902	6.184
Cyanidin	−3.965	0.887	4.852
Resveratrol	−11.581	−4.132	7.449
Gallic acid	−11.026	−3.01	8.016

## Data Availability

Not applicable.
